# A Systematic Survey of Physicians' Insights Into Lipohypertrophy

**DOI:** 10.3389/fpubh.2021.738179

**Published:** 2021-11-23

**Authors:** Min Shen, Yun Shi, Shuai Zheng, Hongqi Fan, Jingjing Xu, Tao Yang

**Affiliations:** Department of Endocrinology and Metabolism, First Affiliated Hospital of Nanjing Medical University, Nanjing, China

**Keywords:** diabetes, questionnaire-based survey, lipohypertrophy, diagnosis-treatment-care processes, physician

## Abstract

**Background:** It is important that physicians be aware of LH. We designed a questionnaire to determine physician awareness, knowledge, and behaviors regarding LH in clinical practice.

**Participants:** A total of 499 questionnaires were completed by physicians in hospitals from 13 cities in Jiangsu Province, China.

**Key Results:** Compared with physicians at tertiary hospitals, significantly fewer physicians at primary hospitals reported awareness of LH and its screening methods. The proportion of resident physicians aware of LH was significantly lower than the proportion of senior physicians. The proportion of physicians who could identify all LH risk factors among the low-GDP group was significantly higher than the high-GDP group. Only 38.7% of doctors could successfully identify all the hazards associated with LH, but more doctors in tertiary hospitals were able to do so compared to those in secondary and primary hospitals. Compared with tertiary hospitals, the proportions of primary and secondary hospitals with management processes were significantly lower. The proportion of doctors who educated patients regarding LH prevention and treatment in primary hospitals was markedly lower than in tertiary hospitals.

**Conclusions:** Overall, physicians have an inadequate understanding of LH, especially in primary hospitals.

## Introduction

Type 1 diabetic mellitus (T1DM) patients and approximately one-third of those with type 2 diabetes mellitus (T2DM) require exogenous insulin treatment. Lipohypertrophy (LH) is the most prevalent and well-recognized local cutaneous complication associated with insulin therapy (1, 2). The prevalence of LH in various studies ranged from 14.5 to 88% (median: 56.6%), with higher frequencies reported in subjects who repeatedly inject insulin in restricted areas (3).

Previous studies primarily focused on the associated risks and clinical impacts of LH. Several factors have been reported to affect LH development, including the period of insulin usage, rotation of insulin injection sites, frequency of needle reuse, and recurrent tissue trauma (4–6). The clinical significance of this complication is not simply cosmetic; it might also influence insulin absorption, leading to poor glycemic control (4, 7–10). Additionally, this complication may result in increased daily insulin doses and higher healthcare costs (4, 11).

Concern regarding LH development has increased among diabetes nurse educators. Insulin delivery recommendations were recently published that underscore the importance of examining injection sites for LH and the need to educate patients to inspect their own injection sites (12). Similar concepts have been stressed in Chinese national guidelines. Despite the release of injection technique guidelines and increased education, the prevalence of LH remains high. Various factors might preclude adherence to these recommendations. Few studies have focused on physicians' insights regarding LH. Physicians play central roles in diabetes management, so it is important that healthcare professionals are adequately aware of the risks of LH.

We developed a questionnaire to survey physicians in Jiangsu Province, China, to assess their insights regarding LH with regard to three aspects: awareness, knowledge, and behaviors in clinical practice.

## Materials and Methods

### Survey Development

The survey consisted of 16 questions and could be completed in 10 min. The questionnaire was concerned with the following subjects: (1) awareness of LH; (2) knowledge of LH, including associated risk factors and clinical impacts; and (3) behaviors of physicians regarding LH in clinical practice. We used trained research staff to assess whether the questionnaire were filled correctly.

### Survey Administration

A cross-sectional survey was employed in the present study. In November 2020, 499 physicians from hospitals in 13 cities in Jiangsu Province, China, were surveyed using the designed questionnaire. The physicians were classified according to their gross domestic product (GDP) per capita, position within the hierarchical medical system, and professional title. After retrieving the questionnaires and excluding non-valid samples with either no answers or conflicting answers, the data were subjected to statistical analysis.

### Statistical Analysis

The study variables were subjected to a descriptive analysis. In this paper, we used the Statistical Package for the Social Sciences (SPSS) version 25.0 to run all statistical analysis. Answers were compared among multiple groups (according to professional titles, regions with different GDPs per capita, or hierarchical medical system) using chi-squared tests. Significance was defined as *P* < 0.05. Multiple comparisons among physicians were analyzed by the Bonferroni correction.

## Results

### A High Response Rate and Effective Rate Were Achieved

**Figure 1:**
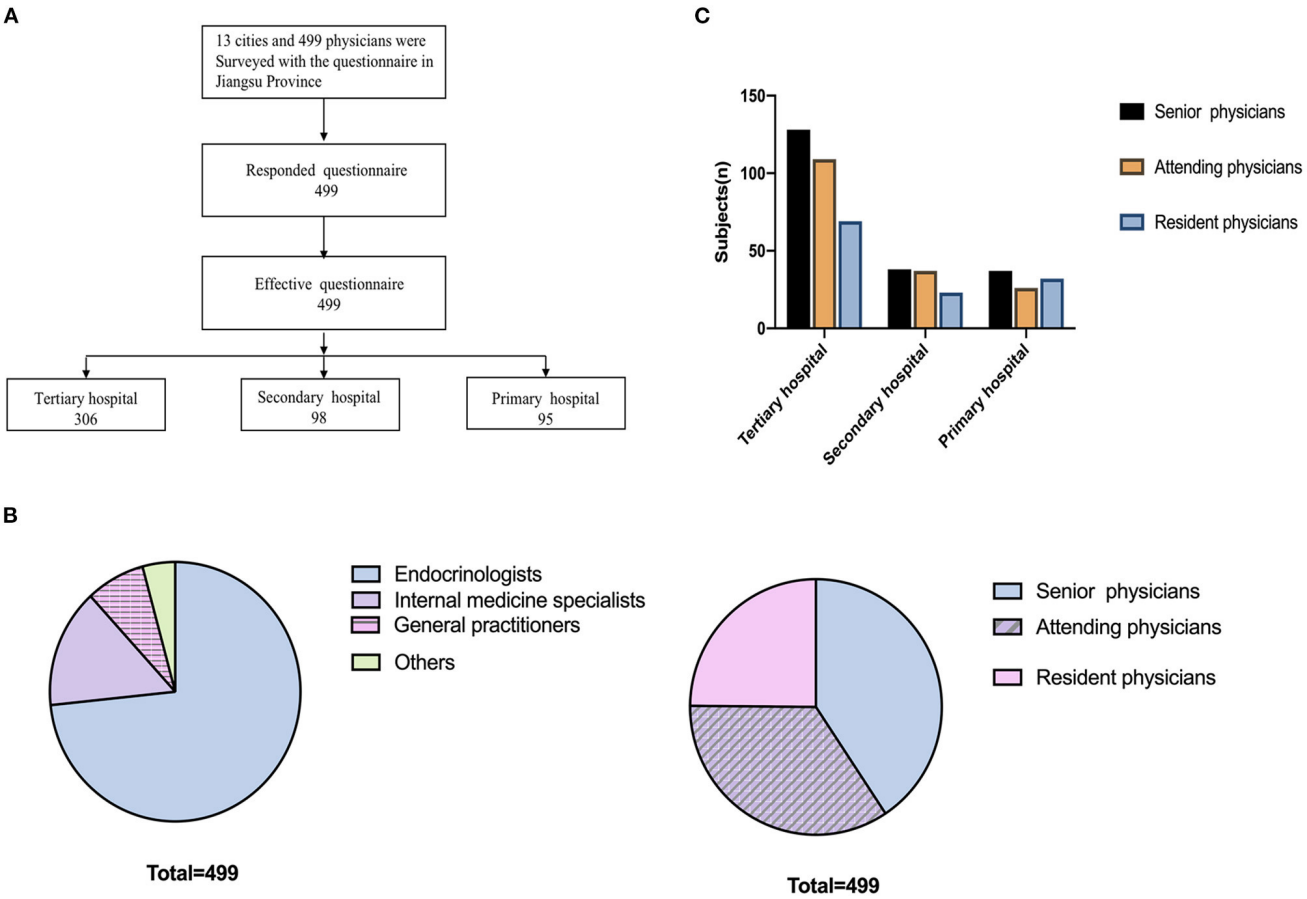
Survey administration. **(A)** 499 physicians in 13 cities of Jiangsu province completed the survey. **(B)** Comparison of the participating physicians. **(C)** Physician seniority composition.

This questionnaire had high retrieval and validity rates (Figure 1A). The surveyed hospitals from the 13 cities of Jiangsu province were distinguished according to the three-tiered system: 306, 98, and 95 physicians worked in tertiary, secondary, and primary hospitals, respectively (Figure 1A). Among the respondents, there were 74 internal medicine physicians, 366 endocrinologists, 38 general practitioners, and 21 other physicians (Figure 1B). The physicians involved in this survey included 203 senior physicians, 172 attending physicians, and 124 resident physicians (Figure 1B). Among them, 11.6% had practiced for ≤3 years, 48.9% for 4–10 years, and 39.5% for >10 years.

The GDPs per capita of seven cities were higher than the average GDP per capita of Jiangsu province (CNY 123.6 thousand in 2019). The ratios of senior to resident physicians at different tiered hospitals were not significantly different (Figure 1C).

### LH Awareness

#### LH Awareness Differed Between Professional Titles and Hospital Grades

**Figure 2:**
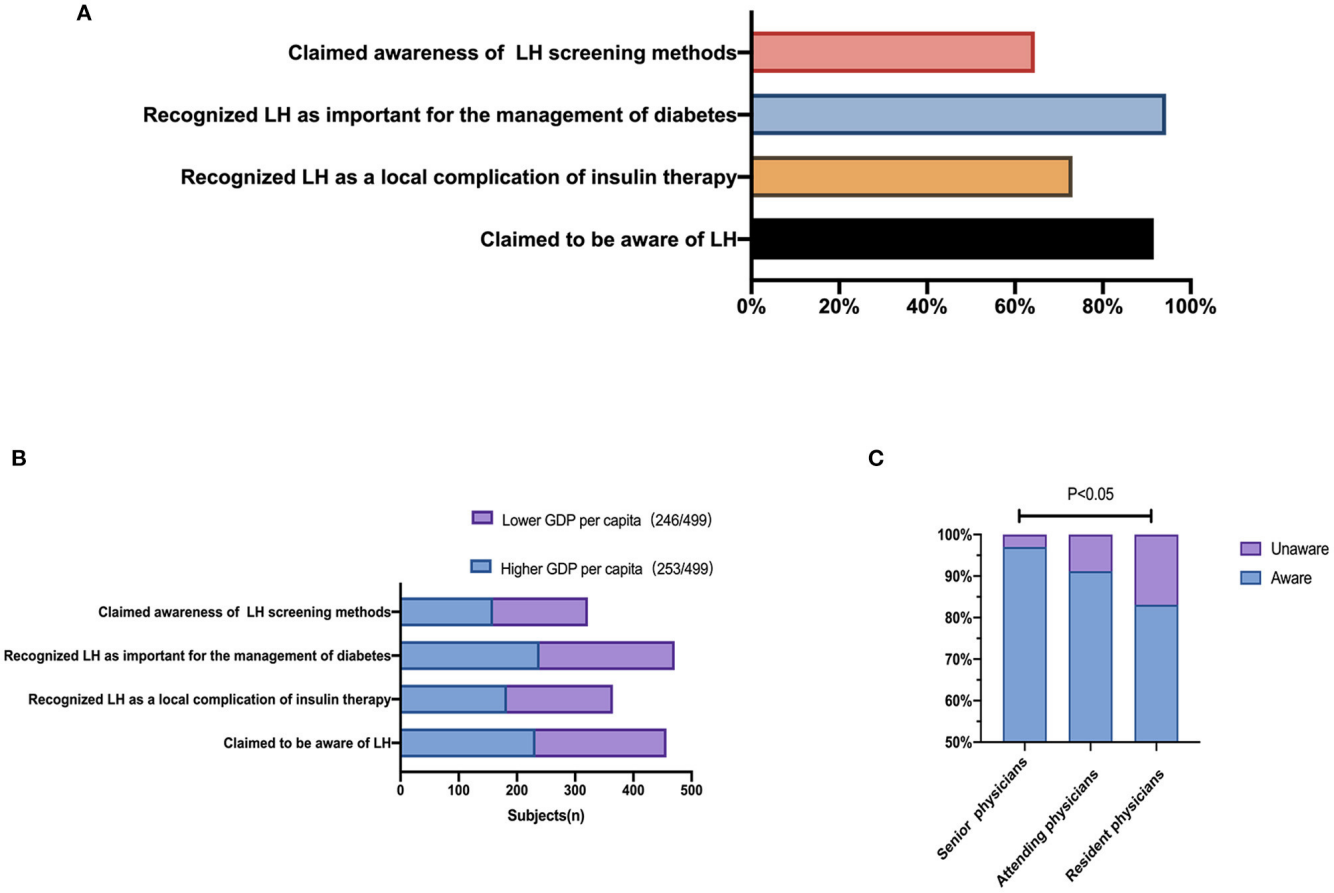
Recognition of lipohypertrophy. **(A)** Proportions of physicians who reported awareness of LH. **(B)** Proportions of physicians aware of LH according to the GDP per capita of the region their hospital serves. **(C)** Proportions of physicians aware of LH according to different professional titles.

The majority (91.6%, 457/499) of physicians claimed that they were aware of LH, and 73.1% (365/499) recognized LH as a local complication of insulin therapy. In addition, 94.4% (471/499) of physicians recognized that LH was important for diabetes management, and 64.5% (322/499) claimed to be aware of LH screening methods (Figure 2A). No significant differences were identified in LH awareness between the high and low GDP per capita regions (*P* > 0.05, Figure 2B). Compared with physicians at tertiary hospitals, significantly fewer physicians at primary hospitals reported being aware of LH and associated screening methods (Table 1). The proportion of resident physicians aware of LH was significantly lower than the proportion of senior physicians (Figure 2C).

**Table 1:** Insights into LH awareness according to the different hierarchical medical systems.

**Hospitals**	**Tertiary**	**Secondary**	**Primary**	***P***
	***n* = 306**	***n* = 98**	***n* = 95**	
**Awareness**				
Claimed to be aware of LH	94.1%	93.9%	81.1%	0.000
Recognized LH as a local complication of insulin therapy	75.5%	67.3%	71.6%	>0.05
Recognized LH as important for diabetes management	95.8%	94.9%	89.5%	0.081
Claimed awareness of LH screening methods	69.9%	59.2%	52.6%	0.004
**Knowledge**				
Associated risk factors (score 5)	40.0%	40.8%	36.8%	0.472
Clinical impacts (score 7)	45.8%	35.7%	18.9%	0.041
**Behavior**				
Management process	82.4%	59.2%	54.7%	0.000
Checked the insulin injection site regularly	79.1%	80.6%	70.5%	0.171
Educated patients about LH	85.9%	84.7%	74.7%	0.041

#### Under Recognition of New LH Screening Techniques

**Figure 3:**
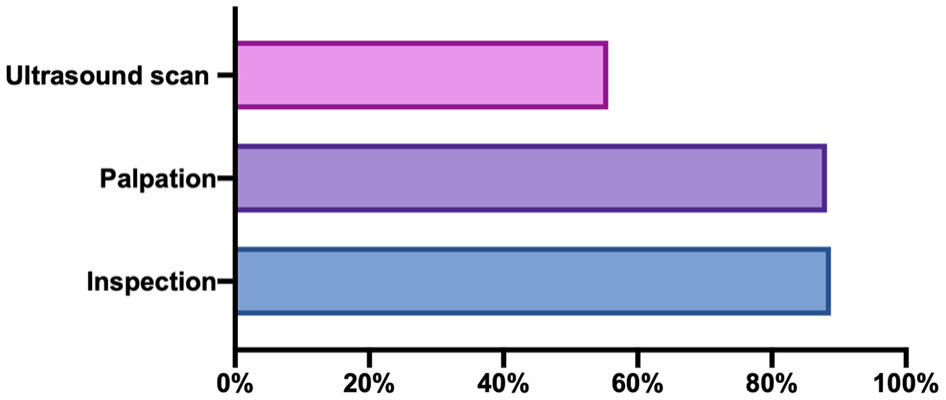
Lipohypertrophy screening method.

More than 80% of the clinical workers indicated that inspection and palpation could be used to screen LH, whereas only 55.6% (179/322) indicated that LH could be screened by ultrasound. A total of 50.3% of the clinical workers answered that inspection, palpation, and ultrasound could be used to identify LH (Figure 3).

### Knowledge About LH

#### Physicians' Understanding of the Associated Risk Factors and Clinical Hazards Associated With LH Is Inadequate and Differs According to Regional and Hospital Level

**Figure 4:**
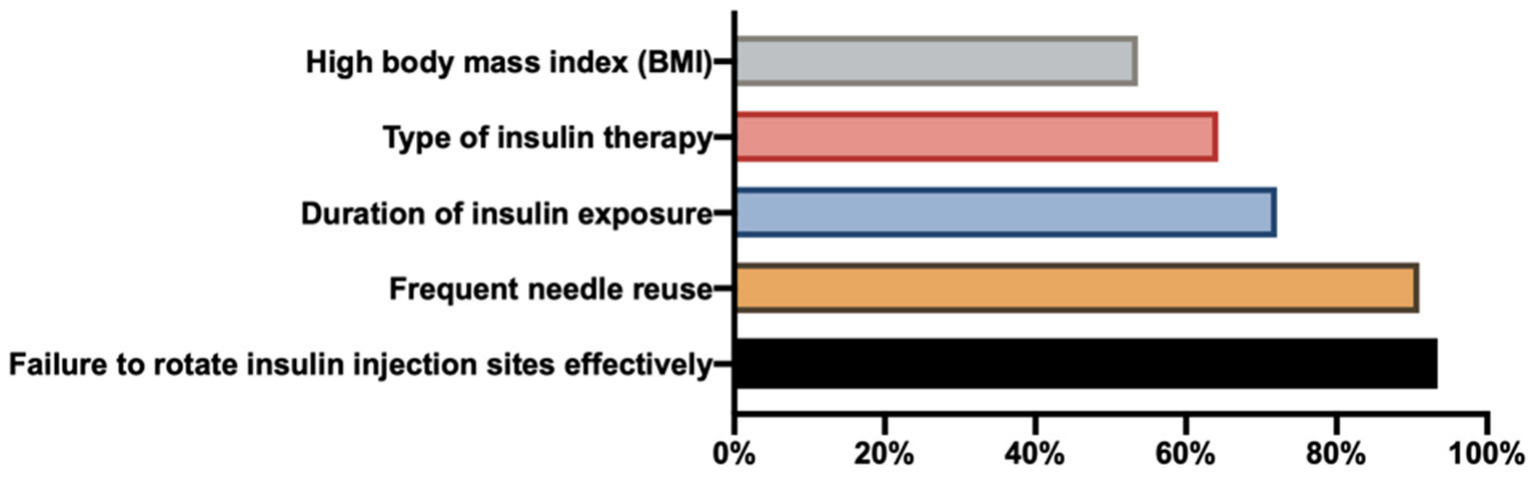
Risk factors associated with lipohypertrophy.

Physicians primarily believe that failure to effectively rotate insulin injection sites and frequent needle reuse are responsible for LH development (Figure 4). We assigned one point to each of the following risk factors: high body mass index (BMI), failure to rotate insulin injection sites, type of insulin therapy, duration of insulin exposure, and frequent needle reuse. Of the physicians, 39.6% (197/498) thought that all five of these factors could contribute to LH occurrence. No significant difference in the proportions of physicians with a score of five was observed among different hospital levels (Table 1). The proportion of physicians in the low-GDP group with a score of five was significantly higher than the proportion in the high-GDP group (50 vs. 29.2%, *P* = 0.000). No significant difference was observed among different professional titles (*P* > 0.05).

**Figure 5:**
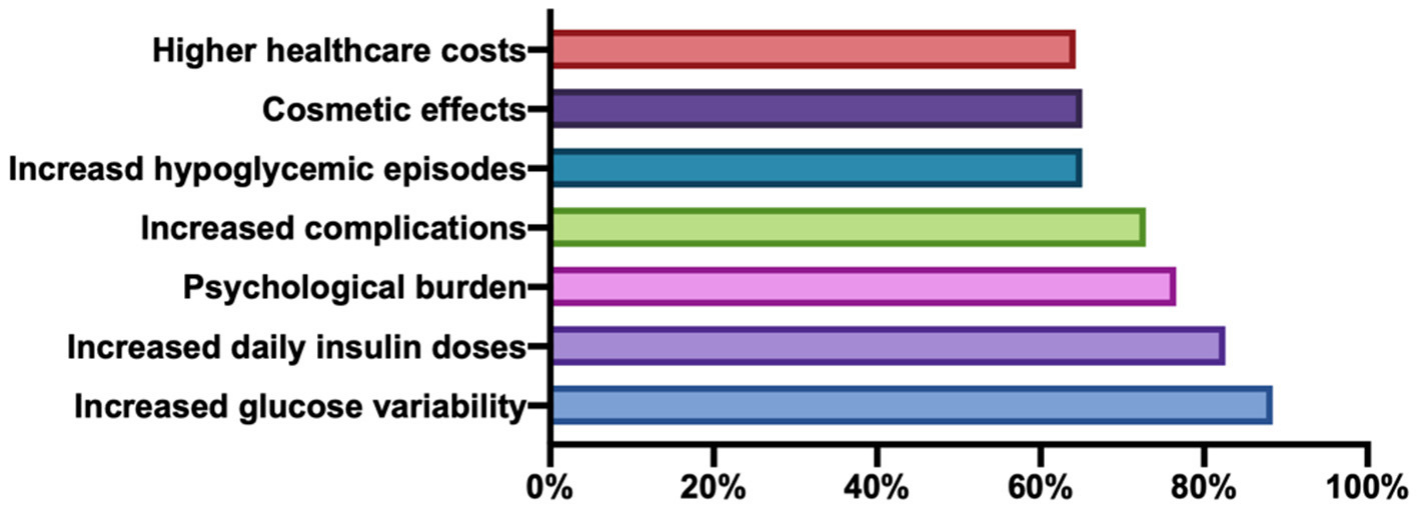
Clinical impacts of lipohypertrophy.

Physicians primarily considered the primary hazards of LH to be greater glucose variability and increased daily insulin doses (Figure 5). We assigned one point to each of the following hazards: increased glucose variability, increased hypoglycemic episodes, more complications, increased daily insulin doses, higher healthcare costs, cosmetic effects, and psychological burden. Only 38.7% (193/499) of doctors considered all seven of these events to be hazards of LH. The proportions of hospitals with a score of seven were significantly different among the three tiers, with more doctors at tertiary hospitals indicating that all seven events were risk factors of LH compared with those at secondary and primary hospitals (Table 1). No significant differences in LH knowledge were identified among the different professional titles or between low- and high-GDP regions.

### Behavior of LH Management

#### Regular Inspections of Insulin Injection Sites and Patient Education Remain Insufficient at Primary Hospitals

Overall, 72.5% (362/499) of physicians indicated that their hospitals had established relevant management process, and there was no significant difference between high- and low-GDP regions (73.9 vs. 71.1%, *P* > 0.05). Compared with tertiary hospitals, the proportions of hospitals with established management processes were significantly lower among the secondary and primary hospitals (Table 1).

Among the surveyed physicians, 77.8% (388/499) claimed to regularly check insulin injection sites, with no significant differences observed between high- and low-GDP areas (77.5 vs. 78.0%, *P* > 0.05), among professional titles (79.3 vs. 76.2 vs. 77.4%, *P* > 0.05), or among hospital grades (79.1 vs. 80.6 vs. 70.5%, *P* > 0.05; Table 1).

A total of 83.6% (417/499) participants stated that they educated patients regarding the prevention and treatment of LH, with no significant difference between high- and low-GDP areas (81.8 vs. 85.4%, *P* > 0.05) or among different professional titles (85.7 vs. 84.3 vs. 79.0%, *P* > 0.05). The proportion of physicians that educated patients in LH prevention and treatment at primary hospitals was markedly lower than at tertiary hospitals (Table 1).

## Discussion

Our survey demonstrated that LH insights of three aspects (awareness, knowledge, and behavior) were inadequate among physicians in China. Most physicians claimed to be aware of LH; however, the level differed among professional titles and hospital grades. Most physicians claimed that LH could be identified by inspection and palpation, and only half of physicians recognized the usefulness of ultrasound screening (a new screening method for LH). Although physicians had an inadequate understanding of the associated risk factors and clinical hazards of LH, it differed according to the region and hospital level. Although a management process was established at most hospitals and most physicians reported regularly inspecting insulin injection sites and educating patients about LH, these protocols appear to be insufficient at primary hospitals.

LH occurs in patients with both T1DM and T2DM and is characterized by the development of a thickened, “rubbery” lesion in the subcutaneous tissue following multiple injections performed at the same site (13). In our survey, most physicians claimed that they recognized LH as a local complication of insulin therapy. However, the results demonstrated the continued existence of insufficient LH awareness.

First, despite the increased importance of LH, no standard methods exist for defining the presence and extension of lipohypertrophic areas, which are not simple processes. Inspection and palpation are common clinical practices used to identify LH (12), but its presence can be underestimated when only these techniques are used. The reliability of this method is potentially low, with high levels of inter-clinician variation recently demonstrated by Gentile et al. (14). Ultrasound scans were recently shown to successfully identify LH with significantly increased frequency compared with inspection or palpation (15). This method can better define the extension of lipohypertrophic areas and identify the nature and severity of LH with greater detail than palpation, enabling more accurate LH grading (size, distribution, and elasticity) (16). Ultrasound is a potentially more objective diagnostic method. In our survey, only half of the physicians recognized the clinical use of ultrasound assessment for LH.

Second, previous studies have described altered insulin absorption rates at LH sites, and LH has been associated with increased glucose variability, poor metabolic control, and increased hypoglycemic episodes (1, 7–10). These outcomes have additional negative impacts on long-term outcomes, including increased daily insulin doses and higher healthcare costs (4, 11), which can have dramatic clinical, social, and economic effects. In our survey, most physicians recognized the clinical harm of LH, but approximately one-third of physicians ignored social and economic costs including increasing healthcare costs, cosmetic effects, and serious psychological burden.

Third, although the cause of LH has not been fully established, known risk factors associated with its development include high BMI, frequent needle reuse, failure to effectively rotate insulin injection sites, and the duration of insulin exposure (2, 5, 6, 10, 17). Indeed, the relationship between BMI and LH is inconsistent. Some studies indicated that higher BMI was a potential risk factor for LH (17, 18), whereas others argued that BMI was not correlated with LH occurrence (2, 10), and a few reported that lower BMI might be an LH risk factor (19). Our previous study also identified higher BMI as a risk factor (20). In our survey, most physicians recognized the associated risk factors, but only ~40% of physicians recognized all of them. Thus, physicians may have difficulty educating their patients to avoid all risk factors.

Hospitals in China are organized according to a three-tiered system (primary, secondary, or tertiary) that recognizes the abilities to provide medical care and education and conduct medical research (21). Primary hospitals are tasked with providing preventive care, minimal health care and rehabilitation services. Secondary hospitals tend to be affiliated with a medium size city, county or district. They are responsible for providing comprehensive health services, as well as medical education and conducting research on a regional basis. Tertiary hospitals are responsible for providing specialist health services, perform a bigger role with regard to medical education and scientific research and they serve as medical hubs providing care to multiple regions (22). Actually, many patients with diabetes and other chronic diseases who live in less-developed regions are treated by physicians from primary or secondary rather than tertiary hospitals. Thus, the professional skills and knowledge of internal medicine physicians should be improved in China. Our survey revealed significant variation in the awareness, knowledge, and behaviors regarding LH across different tiers. At primary hospitals, only 18.9% of physicians clearly recognized the clinical harms associated with LH, and only 54.7% of hospitals had implemented management processes. The proportions of physicians aware of LH and its diagnostic methods were significantly lower in primary hospitals than in the other tiers. Consequently, fewer physicians educated patients regarding the risks of LH. A lack of awareness of LH significance may exist among diabetes professionals at primary hospitals. Although the condition is not life-threatening, it can hamper diabetes management. Thus, increasing LH awareness among physicians; establishing standardized screening, diagnostic, treatment processes; and educating patients regularly are urgently needed at primary hospitals in China. More and more researchers pay much attention to this clinical problems of LH lesions and make efforts. Gentile et al. has demonstrated that structured education led to consistently improved metabolic results, a lower insulin requirement and decreased overall healthcare costs in insulin-treated people with type 2 diabtes (23). Furthermore, repeated refresher courses, at least at 6-month intervals, are needed (24). Thus, due to an inadequate understanding of LH, we should provided education sessions to physicians and investigate the effects.

The strengths of our study are the large sample size and the comprehensive set of questions regarding LH including diagnostic methods, clinical impacts, associated risk factors, and clinical practices. The study also has some limitations. First, the physicians' behaviors regarding LH were self-reported, so some problems could be underestimated due to the reluctance of participants to admit their errors. Second, this study only reports on the experience of Jiangsu Province, and these results cannot be generalized to patients in different regions.

Taken together, our results show that despite some improvements in recent years, LH awareness continues to be suboptimal among many physicians, highlighting the need for comprehensive and continuous education that covers all aspects associated with LH.

## Data Availability Statement

The raw data supporting the conclusions of this article will be made available by the authors, without undue reservation.

## Ethics Statement

The studies involving human participants were reviewed and approved by the Institutional Review Board of the First Affiliated Hospital of Nanjing Medical University. The patients/participants provided their written informed consent to participate in this study.

## Author Contributions

MS initiated and led the project. YS, MS, and SZ analyzed the data. HF collected the questionnaires. JX and TY reviewed and edited the article. All authors contributed to the article and approved the submitted version.

## Conflict of Interest

The authors declare that the research was conducted in the absence of any commercial or financial relationships that could be construed as a potential conflict of interest.

## Publisher's Note

All claims expressed in this article are solely those of the authors and do not necessarily represent those of their affiliated organizations, or those of the publisher, the editors and the reviewers. Any product that may be evaluated in this article, or claim that may be made by its manufacturer, is not guaranteed or endorsed by the publisher.
